# Genetic network identifies novel pathways contributing to atherosclerosis susceptibility in the innominate artery

**DOI:** 10.1186/1755-8794-7-51

**Published:** 2014-08-12

**Authors:** Jody Albright, Pamela M Quizon, Aldons J Lusis, Brian J Bennett

**Affiliations:** 1 Department of Genetics, University of North Carolina, Chapel Hill, 500 Laureate Way, Suite 2303, Kannapolis, NC 28081, USA; 2 Department of Nutrition, University of North Carolina, Chapel Hill, NC 2808, USA; 3 Nutrition Research Institute, University of North Carolina, Chapel Hill, NC 2808, USA; 4 Department of Medicine, David Geffen School of Medicine of UCLA, Los Angeles, CA 90095, USA; 5 Department of Human Genetics, David Geffen School of Medicine of UCLA, Los Angeles, CA 90095, USA; 6 Microbiology, Immunology and Molecular Genetics, David Geffen School of Medicine of UCLA, Los Angeles, CA 90095, USA

**Keywords:** Atherosclerosis, Co-expression network, inflammation

## Abstract

**Background:**

Atherosclerosis, the underlying cause of cardiovascular disease, results from both genetic and environmental factors.

**Methods:**

In the current study we take a systems-based approach using weighted gene co-expression analysis to identify a candidate pathway of genes related to atherosclerosis. Bioinformatic analyses are performed to identify candidate genes and interactions and several novel genes are characterized using *in-vitro* studies.

**Results:**

We identify 1 coexpression module associated with innominate artery atherosclerosis that is also enriched for inflammatory and macrophage gene signatures. Using a series of bioinformatics analysis, we further prioritize the genes in this pathway and identify *Cd44* as a critical mediator of the atherosclerosis. We validate our predictions generated by the network analysis using *Cd44* knockout mice.

**Conclusion:**

These results indicate that alterations in *Cd44* expression mediate inflammation through a complex transcriptional network involving a number of previously uncharacterized genes.

## Background

Cardiovascular disease (CVD) is the leading cause of death in the United States and the incidence of CVD is rapidly increasing in other countries [1]. A common cause of cardiovascular disease is atherosclerosis, a pathological process resulting from an inflammatory response to lipids deposited in the artery wall. Atherosclerosis is a highly complex disease involving interactions among numerous genetic and environmental factors [2]. Well established risk factors for atherosclerosis include: gender, race, dyslipidemia, diabetes and a family history of disease; however these factors alone do not account for differences in susceptibility to atherosclerosis. Based on human postmortem studies as well as experimental studies in model organisms, it appears that these systemic risk factors act in part by affecting local inflammation in the vessel wall [3-5].

Inflammation is a fundamental biological process that provides protection from infection, but when dysregulated, acts as a source of chronic disease. In particular, there has been considerable interest in the role of inflammation in the etiology of atherosclerosis [6,7] and improving our understanding of how inflammatory pathways affect atherosclerosis may identify new therapeutic targets. Unfortunately, understanding how to regulate the immune system to reduce chronic disease- associated inflammation has been difficult. Monocyte-derived cells, such as macrophages and dendritic cells, are critical to immune system function and are intimately involved in atherosclerosis [8-10]. These cells along with lymphocytes are found at sites of inflammation and within atherosclerotic lesions [11]. Macrophages are of particular interest because macrophage deficiency prevents atherosclerotic lesion formation in mice [12,13] and because macrophage emigration from lesions leads to reduced lesion size [14]. Thus, identification of pathways that regulate vascular inflammation and macrophage function may provide novel therapeutic targets.

We previously published Quantitative Trait Locus (QTL) studies that identified a novel locus controlling innominate artery (IA) lesion size, with a 95% confidence interval spanning 14 Mb on Chromosome 2. These studies used F2 progeny from a genetic cross between C57BL6/J.Apoe^-/-^ mice, classically characterized as atherosclerosis susceptible, and C3H/HeJ.Apoe^-/-^ mice, classically characterized as atherosclerosis resistant [15]. Surprisingly, this QTL was not identified to regulate atherosclerosis in the aortic sinus of mice from the same cross [16], indicating a unique genetic contribution among various vascular sites. Furthermore, the susceptible allele for this QTL was derived from the C3H/HeJ mice, the strain historically characterized as atherosclerosis resistant [16-18]. Among the 360 genes within the QTL, we identified *Cd44* as a high probability candidate gene for this QTL based on its physical location within the QTL boundary, the high correlation between IA atherosclerosis and the mRNA levels of *Cd44*, and prior reports of its role in atherosclerosis [19,20] and inflammation [21]*.* However, the gene(s) within the QTL responsible for the increased atherosclerosis susceptibility and more importantly a mechanism for the increased susceptibility remains unknown.

The primary objective of this study was to identify novel pathways and mechanisms contributing to innominate artery atherosclerosis. Using Weighted Gene Co-Expression Network Analysis (WGCNA) we identify a module (group) of highly related transcripts, correlated with IA lesion size. This module is enriched with genes normally expressed in macrophages suggesting either the influence of Kupfer cells in the liver or general alteration of tissue macrophage response to atherosclerotic stimuli. We characterize the expression of several of the genes in this module through cell culture experiments using primary macrophages. Causal modeling using Network Edge Orienting analysis confirm *Cd44* as a likely causal gene within this pathway. We also identify several key genes within the module that are sensitive to altered *Cd44* expression and likely to affect atherosclerosis risk.

## Methods

### Quantitative trait locus studies

QTL results have been previously reported [15]. In brief, C57BL/6J.Apoe^-/-^ mice were purchased from The Jackson Laboratory and C3H/HeJ.Apoe^-/-^ mice were bred by backcrossing B6.Apoe^-/-^ to C3H/HeJ for 10 generations. F2 mice (BxH Apoe^-/-^) were generated by crossing B6.Apoe^-/-^ with C3H.Apoe^-/-^ and subsequently intercrossing the F1 mice as described [16]. The F2 mice (n = 86) mice were fed a Western diet (Teklad 88137) containing 42% fat and 0.15% cholesterol for 16 weeks until euthanasia and innominate artery phenotyping at 24 weeks of age. A genetic map with markers about 1.5 cM apart was constructed using SNP markers as described [16]. RNA was isolated from tissues of the F2 mice using Trizol and microarray analysis was performed on the RNA using 60mer oligonucleotide chips (Agilent Technologies) as previously described [22]. Expression data can be obtained from GEO databases for liver (GSE2814).

### Weighted gene co-expression network analysis

Network analysis was performed using the WGCNA R package [23]. An extensive overview of WGCNA, including numerous tutorials, can be found at http://labs.genetics.ucla.edu/horvath/CoexpressionNetwork/Rpackages/WGCNA/ and this method has been extensively used to create co-expression networks [23-28]. To begin, we filtered the array data to include 8173 probes expressed in the liver as previously described [29]. To generate a co-expression network for the selected probes, an adjacency matrix is created by first calculating the pairwise gene:gene correlations for all 8173 probes and then raising the Pearson correlation to the 8th power. The power was selected using the scale-free topology criterion, which is determined by the function “pickSoftThreshold” in the WGCNA package [23,30]. Network connectivity (k.total) of the genes was calculated as the sum of the connection strengths with all other network genes. A TOM-based dissimilarity measure was used for hierarchical clustering of the genes. Gene modules corresponded to the branches of the resulting dendogram and were defined using the “Dynamic Hybrid” branch cutting algorithm [31]. The parameters for module generation were as follows: “cut height” parameter was set to 0.97 and the “minimum module size” parameter was set to 50. Gene significance (GS) for each gene was determined and is defined as the correlation between innominate artery atherosclerosis and expression of probes. Module significance (MS) was calculated as the mean GS for all module genes. Module eigengenes were defined as the first principal component calculated using PCA. Overall network visualization and sub-networks were visualized using Cytoscape [32].

### Gene ontology

We performed a Gene Ontology (GO) enrichment analysis for network modules using the Database for Annotation, Visualization and Integrated Discovery (DAVID) using the functional annotation clustering option [33]. Functional annotation clustering combines single categories with a significant overlap in gene content and then assigns an enrichment score (ES; defined as the –log^10^ of the geometric mean of the unadjusted P-values for each single term in the cluster) to each cluster.

### Causality modeling

Causal relationships were identified using Network edge orienting (NEO) as previously described [34]. An R package is available at http://labs.genetics.ucla.edu/horvath/aten/NEO/ and assigns direction to the edges of a network using structural equation models that integrate genetic markers, gene expression levels and clinical traits. NEO estimates the probability of 3 models: causal, reactive and independent. We restricted the analyses to the peak marker of the Chr 2 QTL for innominate artery atherosclerosis, individual gene expression levels for genes in the brown module, and size of innominate artery atherosclerosis. We used the single marker analysis function of NEO (LEO.NB.SingleMarker) score, which is the log10 probability of this model divided by the log10 probability of the next best fitting alternative model [34]. LEO scores in excess of 0.3 were set as a threshold of for further investigation as a value of 0.3 indicates that the causal model is two times more likely, given the data, then the next best model, similar to previous studies [24,35]. The Root Mean Square Error of Approximation (RMSEA) is an index of model fit and was used to evaluate the overall model fit for NEO. RMSEA indices close to zero indicate good model fit and a threshold of 0.05 was used as previously suggested [34].

### Cell culture studies

C57BL/6J, C3H/HeJ and *Cd44*^-/-^ mice where purchased from the Jackson Laboratory and bred at the David Murdock Research Institute. Mice were handled in strict accordance with the recommendations in the Guide for the Care and Use of Laboratory Animals of the National Institutes of Health under protocols approved by the Institutional Animal Care and Use Committees of the North Carolina Research Campus (Protocol Number: 12–003). Mouse peritoneal macrophages where isolated from C57BL/6J, C3H/HeJ and *CD44*^-/-^ four days after an IP injection of thioglycolate as previously described [36]. Cells were washed with PBS, red blood cells lysed using ACK lysis buffer, and the remaining counted and plated at 100,000 cells per cm^2^ into 12 well plates. Cells were grown in DMEM (Hyclone, Logan, Utah) supplemented with 20% FBS (Hyclone, Logan, Utah) overnight, rinsed with PBS and then adherent cells treated in 1% FBS alone as a control or stimulated with LPS (List Biological Inc., Campbell, CA, #201). Cells were treated for 4 hours except for time-course experiments. RNA was extracted using a Maxwell instrument (Promega, Madison WI) and RNA quality was assessed using an Experion Bioanalysis system (BioRad, Hercules CA).

### Real time PCR

Total RNA was isolated according to manufacturer’s specifications using Promega’s Maxwell 16 with the Maxwell 16 Cell LEV Total RNA Purification Kit (Promega, AS1225). cDNA was synthesized using Applied Biosystems High Capacity cDNA Reverse Transcription Kit (Life Technologies, 4368813). qPCR was done on a Roche Lightcycler 480 II using Kapa SYBR FAST Master Mix (KAPA, KK4609). Relative gene expression was determined using an efficiency corrected method, and efficiency was determined from a 3-log serial dilutions standard curve made from cDNA pooled from all samples. Primers were designed across exon-exon boundaries using Roche UPL guidelines. Results were normalized to *Rpl4*.

### Repository data

Publically available micro-array data (*GEO10000),* which contained replicate samples of aortas from C57BL/6J mice as well as *Apoe*^*-/-*^ mice at 6 weeks and 36 weeks of age was downloaded from gene expression omnibus*.* We matched the genes in our network and this dataset by Entrez gene IDs, and assigned probes to the corresponding module. We examined the expression of our candidate genes in human endarterectomy samples using publically available micro-array data GSE43292. In brief, carotid endarterectomy collected in 32 hypertensive patients. The samples contained media and neo-intima without adventitia. They were paired, including for each patient one sample of the atheroma plaque (stage IV and over of the Stary classification [37]) containing core and shoulders of the plaque, and one sample of distant macroscopically intact tissue (stages I and II).

### Statistical analysis

Statistical analysis was performed using Prism Graphpad software (V5.0). Comparison between control and treatment group(s) was carried out using either a Student’s t test or one-way ANOVA, and statistical significance is shown as described in the figure legends.

## Results

### Transcriptional network analysis identifies pathway contributing to atherosclerosis

**Figure 1:**
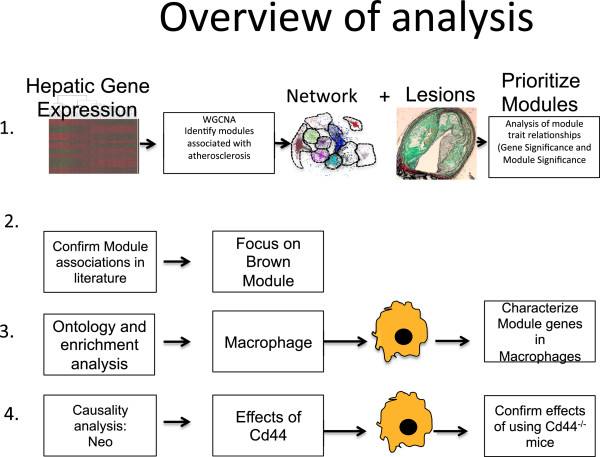
**Overview of analysis: This flowchart presents a brief overview of the analysis and subsequent experiments performed.** 1. Construction of the weighted gene Co-expression network Analysis and relationship to innominate artery atherosclerosis. 2. Relationships between modules and atherosclerosis were confirmed using independent and publically available gene expression datasets. 3. Ontology analysis was performed using DAVID and identified macrophages as a potential cell type for validation of the network. In-vitro experiments are performed to characterize module genes. 4. Causality analysis is performed and experiments using macrophages from gene targeted mice are used to validate predictions.

QTL studies have demonstrated the genetic complexity of atherosclerosis; however, actual identification of the causal gene(s) underlying these QTL is difficult. The difficulty validating candidate genes identified in QTL studies is primarily due to the poor resolution of the approach, large regions of chromosomes are identified and often contain hundreds of genes [38]. For example, the novel locus on Chromosome 2 controlling atherosclerosis development in the IA contains 360 genes [15]. We used global hepatic gene expression, from microarrays, and a network-based approach to further interrogate the effect of this locus. These expression data have previously been used to identify co-expressed genes regulating bodyweight and the metabolic syndrome [39]. Our current study used a subset of the mice (n = 89) that were phenotyped for innominate artery atherosclerosis, an artery that recapitulates human atherosclerosis [40]. The current study uses a series of analytical analyses and cell culture experiments to further interrogate this locus and an overview of the analyses and experiments is shown in Figure 1.

**Figure 2:**
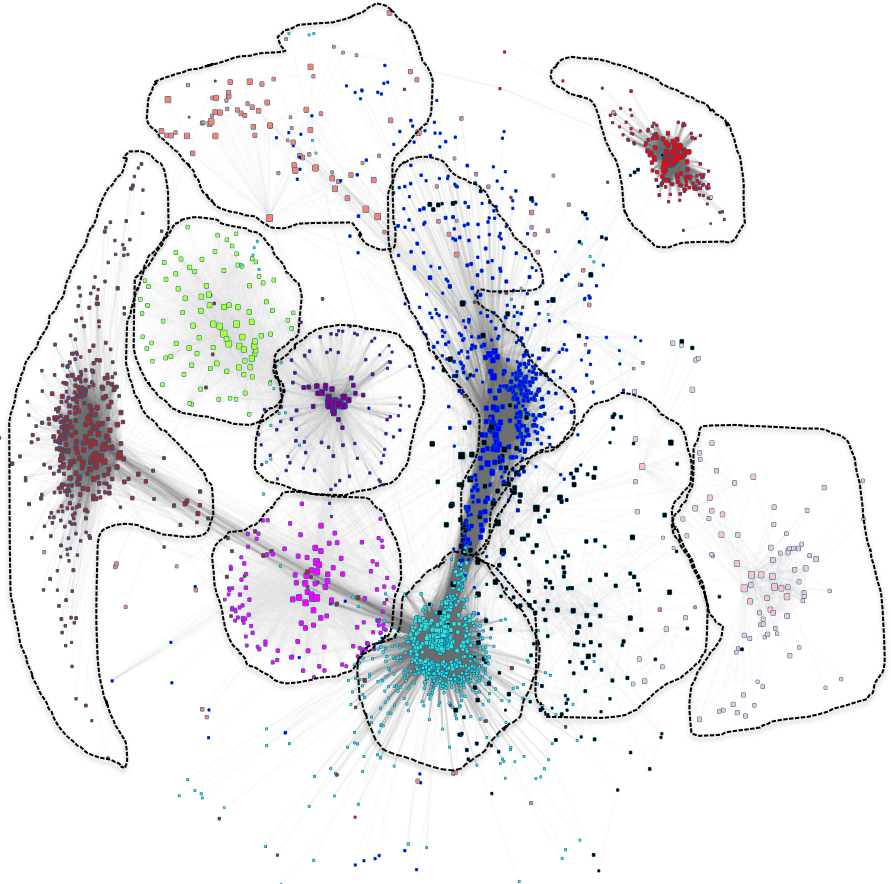
**Genetic network associated with innominate artery atherosclerosis.** Weighted Gene Co-expression analysis of liver RNA identifies 10 modules of highly co-expressed genes. Networks are visualized in Cytoscape. Note size of the module denotes the overall connectivity of the hubs and strength of the topological overall is denoted by the length of the edge.

We constructed a co-expression network by grouping the expression of genes in the liver based on their topologic overlap, using Weighted Gene Co-expression Network Analysis (WGCNA) [23]. To perform the current analysis, we selected 8173 probes representing 8023 unique genes as previously described [22], from the microarray data. We implemented WGCNA with stringent parameters which resulted in 4485 genes being divided into 10 co-expression modules (groups of co-expressed genes) while the remaining 3538 genes were not able to form a module and have not been further analyzed (Additional file 1: Table S1). The hierarchical clustering of the genes into modules is shown in Additional file 2: Figure S1 and the topological overlap of the modules in Additional file 2: Figure S2. The overall network structure was visualized in Cytoscape [32] (Figure 2).

We next performed enrichment analysis using DAVID [33] and found that eight of the 10 modules were significantly enriched with genes representing specific Gene Ontology (GO or Kyoto Encyclopedia of Genes and Genomes (KEGG) classifications (Table 1). A list of each gene contained in the 10 modules is provided in Additional file 1: Table S1 and all enrichment categories for the modules are provided in Additional file: 3 Table S2.

**Table 1:** Characterization of modules

**Module number**	**Module**	**Number of probes**	**Number of unique genes**	**Enrichment term**	**Fold enrichment**	**FDR**
**1**	Black	726	711	IPR001909:Krueppel-associated box	3.97	1.39×10^-07^
**2**	Blue	862	847	IPR017973:Cytochrome P450, C-terminal region	4.9	3.93×10^-04^
**3**	Brown	666	655	GO:0001817 ~ regulation of cytokine production	4.41	1.64×10^-08^
**4**	Green- yellow	96	94	SP_PIR_KEYWORDS: protein transport	5.45	4.15×10^-04^
**5**	Magenta	134	132	SP_PIR_KEYWORDS: protein transport	5.45	4.15×10^-04^
**6**	Pink	173	167	SP_PIR_KEYWORDS: methyltransferase	6.32	0.044
**7**	Purple	115	111	GO:0030529 ~ ribonucleoprotein complex	5.29	0.012
**8**	Red	315	315	none		
**9**	Salmon	524	517	GO:0005578 ~ proteinaceous extracellular matrix	3.1	2.32×10^-06^
**10**	Turquoise	946	936	GO:0007186 ~ G-protein coupled receptor protein signaling pathway	2.3	8.39×10^-10^
**No module**	Grey	3616	3538			

**Figure 3:**
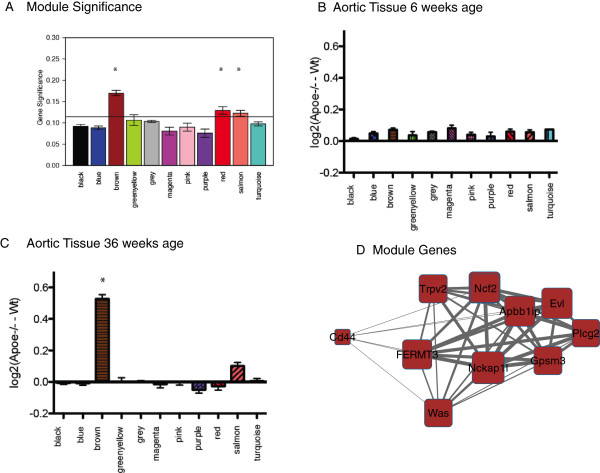
**Co-expression Network analysis identifies the Brown module as related to Atherosclerosis. (A)**. Mean MS score for each of the 11 network modules. **(B)** Mean module gene expression in atherosclerotic aorta tissue relative to non-atherosclerotic tissue at 6 weeks. **(C)** Mean module gene expression in atherosclerotic aorta tissue relative to non-atherosclerotic tissue at 36 weeks. In panels **(B–C)** expression is presented as the mean log2 expression for each gene in a module in aorta from C57BL/6 J *Apoe*^-/-^ mice minus log2 expression from wild type C57BL/6 J mice. **(D)** A sub-network of co-expressed genes in the Brown Module including *Cd44* and the brown module hub genes. Gene connectivity determines size of each node. Distance between nodes is determined by the topological overlap. * denotes significant differences (p < 0.05).

While the network was based on hepatic gene expression, the Chr 2 locus was not associated with plasma lipid levels [15]; Thus, we hypothesized that our network analysis would identify novel, non-lipid mediated, pathways associated with lesion development. To determine which genes and modules were related to lesion size, we calculated two metrics, the *Gene Significance* (*GS)* and *Module Significance* (*MS).* The GS is the absolute value of the correlation (Pearson’s r) between the expression of each gene in our network and the extent of lesion development in the innominate artery [23] and is used to identify genes that are related to lesion size. The MS is the mean GS for each module [23] and is used to identify which modules are most highly correlated with lesion size. To determine the significance threshold for the *MS* metric we created 10000 sets of 400 *GS* values and calculated the correlation with IA atherosclerosis. Using a 1-sided distribution of 0.95 we found that a *MS* of 0.15 corresponds to a p < 0.05. From this, we identified 3 modules that exceeded this threshold, (brown, red and salmon Figure 3A), and so were significantly related to IA lesion size.

We next hypothesized that our atherosclerosis-related network modules should contain genes that are over-represented in atherosclerotic tissue. To perform this analysis we used publically available microarray data (*GEO10000),* which contained replicate samples of aortas from C57BL/6J mice as well as *Apoe*^*-/-*^ mice at 6 weeks and 36 weeks of age*.* We matched the genes in our network and this dataset by Entrez gene IDs, and assigned probes to the corresponding module. Of the 4485 genes in our network, this array contains 3992 genes, represented by 8205 probes. These probes were assigned to the corresponding network modules and the fold change in expression was calculated by subtracting the mean log_2_ expression values of the C57BL/6 J mice from mean log_2_ expression values of the *Apoe*^*-/-*^ mice. At 6 weeks of age, when lesion size was expected to be small, there was no enrichment of any of module (Figure 3B). We repeated this analysis at 36 weeks of age, a time when IA lesion size was developed in the *Apoe*^-/-^ mice, and found that only 1 module, (the brown module), contained significant enrichment of genes overexpressed in atherosclerotic tissues (Figure 3C). The co-expression pattern of the Brown Module genes discussed in detail in the current manuscript are shown in Figure 2D.

We again used DAVID [33], to search for tissues and cell types in which these genes were over-represented and found that the brown module is enriched for genes expressed in activated spleen and macrophages (p < 8.01×10^-10^ and p < 2×10^-06^, Bonferroni corrected) Table 1. We confirmed the macrophage specificity of this module by examining the enrichment of the core macrophage signal recently identified by the Immunological Genome (ImmGen) Project [41]. Of the 39 genes comprising the core macrophage transcriptional profile, 26 were assayed in our microarray studies and 13 were enriched in the brown module (p < 3.3×10^-11^, Fisher’s exact test). The enrichment of other modules was calculated using DAVID and is shown in Additional file 3: Table S2.

### Candidate gene identification in the brown module

We next focused on identifying candidate genes in the brown module that regulate the response to atherosclerotic and inflammatory stimuli. Among the 655 genes in this module, several criteria were used to identify candidate genes. Our first criterion was to prioritize genes by their connectivity because highly connected genes, also called hub genes, have been previously shown to be key regulators of the module [29,42,43]. In particular, connectivity and GS are significantly correlated in the brown module (Additional file 2: Figure S3), further indicating that identifying highly connected genes will yield candidates with a strong relationship to atherosclerosis. The most connected genes in this module are: *Nckap1l, Evl, Apbb1ip, Fermt3, Ncf2, Trpv2, Gpsm3, Was, Plcg2* (Table 2). Our positional candidate, *Cd44,* was not a highly connected gene ranking 362 out of the 666 genes in the module (Additional file 1: Table S1).

**Table 2:** Hub Genes in brown module

**Gene symbol**	**Gene name**	**Chr**	**Mb**	**Kme**	**Expression regulated by** **Chr 2: eQTL**	**Role in atherosclerosis**	**Role in monocyte derived cells**	**Known candidate for human disease**	**Leo score**	**RMSEA**
**Nckap1l (Hem-1)**	NCK associated protein 1 like	15	103	1	Yes (6.18)	None Known	Yes [44]	None Known	-1.22	0.255
					105.89 Mb					
**Evl**	Ena-vasodilator stimulated phosphoprotein	12	108	0.93	Yes (6.17)	None Known	None	leukocyte adhesion deficiency	-1.46	0. 280
					84.17 Mb					
**Apbb1ip (RIAM)**	amyloid beta (A4) precursor protein-binding, family	2	23	0.92	Yes (7.86)	None Known	Yes phagocytosis [45]	None Known	-21.27	0. 255
					81.80 Mb					
**Fermt3**	fermitin family homolog 3	19	7	0.88	Yes (7.73)	None Known	Yes [46]	leukocyte adhesion deficiency	-0.49	0.223
					105.89 Mb					
**Ncf2**	neutrophil cytosolic factor 2	1	153	0.85	Yes (4.23)	None Known	Yes [47]	Chronic granulomatous disease	-1.87	0.283
					84.17 Mb					
**Trpv2**	transient receptor potential cation channel, subfamily V, member 2	11	63	0.83	Yes (8.19)	None Known	Yes-cell death in response to oxidized LDL [48,49]	None Known	-1.38	0.275
					148.90 Mb					
**Gpsm3**	G-protein signaling modulator 3	17	34	0.82	Yes (5.81)	None Known	Yes [50]	None Known	-1.76	0.28
					105.89 Mb					
**Was**	Wiskott-Aldrich syndrome homolog	X	8	0.81	none		Yes [51]	Wiskott-Aldrich syndrome	-1.33	0.268
**Plcg2**	phospholipase C, gamma 2	8	117	0.80	Yes (7.38)	None Known	Yes [52,53]	Familial cold autoinflammatory syndrome	-0.98	0.254
					105.89 Mb					
**Cd44**	CD44 antigen	2	102	0.26	Yes	Yes	Yes	None Known	1.01	0.000

Kme- is the weighted connectivity for the gene. LEO.NB, log10 ratio of causal model to all other model probabilities; -log10*P*, RMSEA, a model fit index (index close to 0 indicates good fit of the causal model).

### Hub Gene Expression altered by inflammatory stimulus

Atherosclerotic lesions contain many cell types and the over-representation of the brown module genes in macrophages and activated spleen prompted us to attempt to characterize the brown module genes using *in vitro* systems. We first characterized expression of our key brown module genes in thioglycolate-elicited peritoneal macrophages in the basal state and treated with varying amount of *bacterial lipopolysaccharide (LPS)*. We used *Tnf* expression as a positive control for these experiments to demonstrate induction of an inflammatory response. We repeated these experiments with doses of *LPS* between 2 and 200 ng/ml. We observed significant induction of *Apbb1ip, and Cd44* while *Evl*, *Fermt3, Gpsm3 and Was* were down-regulated by increasing amounts of *LPS* (Additional file 2: Figure S4)*.* We also examined the time course response for the brown module genes for treatment times ranging from 2 to 8 hours, and observed that these genes are responsive to *LPS* in a time-dependent manner (Additional file 2: Figure S5). These experiments were also conducted in RAW 264.7 cells with similar results (data not shown).

### Hub gene expression varies by strain

**Figure 4:**
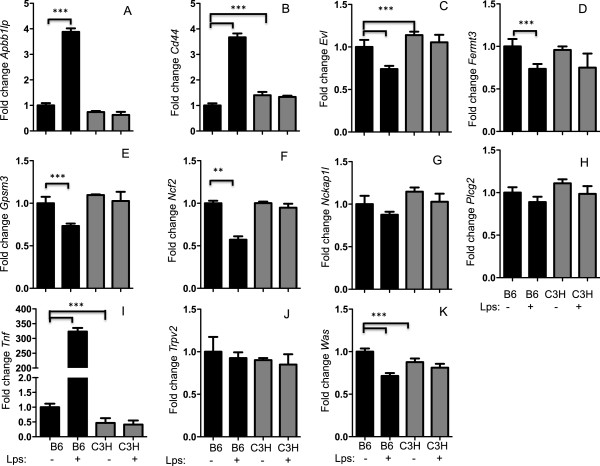
**Strain specific expression of hub genes.** Peritoneal macrophages were isolated from C57BL/6 J and C3H/HeJ mice and treated in triplicate with media or media with 10 ng/ml *LPS* for 4 hours. *Expression of Apbb1ip, Cd44, Evl, Fermt3, Gpsm3, Ncf2, Nckap1l, Plcg2, Tnf, Trpv2,* and *Was* were normalized to *Rpl4* and expressed relative to non-stimulated cells **(Panels A-K respectively)**. * indicates significant differences P < 0.05. Values represent mean ± sem.

The original QTL was found in a cross between C57BL/6 J and C3H/HeJ mice. We therefore next sought to determine expression level of our key candidate genes in peritoneal macrophages from both of these strains with and without the addition of 10 ng/ml *LPS*. We found that *Apbb1ip, Cd44, Evl, Fermt3, Gpsm3, Ncf2, Nckap1l*, *Plcg2, Trpv2,* and *Was* were differentially expressed between strains in unstimulated cells (Figure 4). As expected there was no response in C3H/HeJ to *LPS* as these mice are defective in *Tlr4* signaling (Figure 4).

### Characterizing transcriptional regulation of the brown module

Our WGCNA analysis groups the genes by topological overlap and develops an undirected network with a module (Brown module) of highly connected genes that is associated with innominate artery atherosclerosis. We previously reported an expression QTL (eQTL) for *Cd44*[41] and hypothesized that additional genes in the brown module may be regulated by the Chr 2 locus *in trans* (distant eQTL)*.* Several of our hub genes *Nckap1l*, *Fermt3, Trpv2, Gpsm3* and *Evl*, have eQTL that map to the Chr 2 locus (distant eQTL) Table 2 and http://systems.genetics.ucla.edu/data[54]. The existence of several distant eQTL indicates that a common genetic variant at Chr 2 is regulating the expression of these genes. However, we have not assessed the relationship among the genes in the Brown module nor how transcriptional changes to specific transcripts may alter expression of the Brown module.

To better assess relationship between individual genes in the Brown module and atherosclerosis, we utilized Network Edge Orienting (NEO), a freely available R package [34], to assess the causal relationships between the peak SNP associated with IA lesion size, rs368994, the hepatic expression of genes contained in the Brown module and IA atherosclerosis. This analysis showed that *Cd44* had the highest LEO score indicating that it is the brown module gene with the highest likelihood of a causal relationship with atherosclerosis (Table 2). No other genes in the Brown module had a significant LEO score indicating that differences in *Cd44* expression are proximal to atherosclerosis as compared to the remaining Brown module Genes. Thus, we hypothesized that perturbations in *Cd44* expression may affect the gene expression of genes within the Brown module.

**Figure 5:**
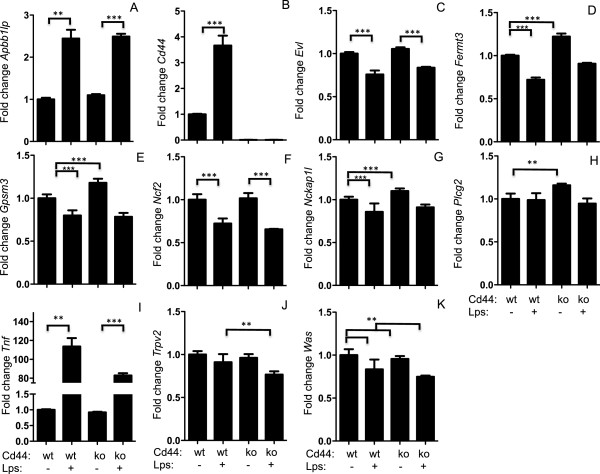
**Cd44 modulates brown module hub gene expression.** Peritoneal macrophages were isolated from C57BL/6 J and *Cd44*^*-/-*^ mice and treated in triplicate with media or media with 10 ng/ml LPS for 4 hours. Expression of *Apbb1ip, Cd44, Evl, Fermt3, Gpsm3, Ncf2, Nckap1l, Plcg2, Tnf, Trpv2,* and *Was* were normalized to *Rpl4* and expressed relative to non-stimulated cells from C57BL/6 J mice **(Panels A-K respectively)**. Genotype of the cells and treatment condition are indicated below the x-axis * indicates significant differences P < 0.05. Values represent mean ± sem.

To investigate the role of *Cd44* on the expression of these module genes, we isolated thioglycolate-elicited peritoneal macrophages from *Cd44*^*-/-*^ and wild-type mice, on a C57BL/6J mice genetic background, and performed qPCR. We first compared the expression of the Brown module genes in unstimulated macrophages and observed small but statistically significant differences in *Fermt3, Gpsm3 Nckap1l*, and *Plcg2.* We also determined the response to *LPS* in both *Cd44*^*-/-*^ and WT macrophages and observed significant differences in *Cd44, Fermt3, Trpv2* and *Was* in response to *LPS* (Figure 5). These results indicate that *Cd44* expression is upstream of the Brown module hub genes and is altering expression of these genes.

**Figure 6:**
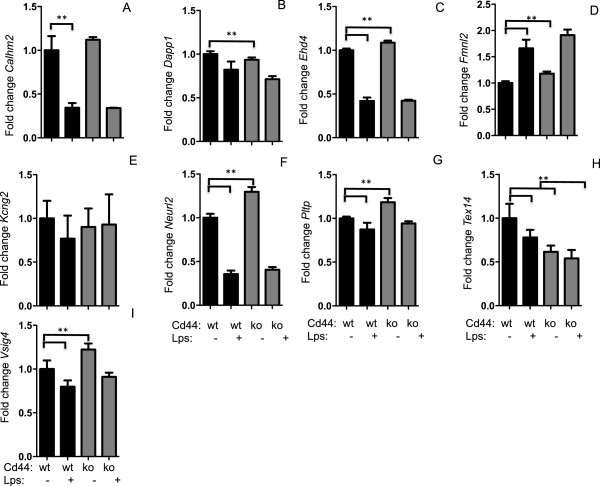
**Confirmation of novel Cd44 target genes.** Peritoneal macrophages were isolated from C57BL/6 J and *Cd44*^*-/-*^ mice and treated in triplicate with media or media with 10 ug/ml *LPS* for 4 hours. Expression of *Calhm2, Dapp1, Ehd4, Fmnl2, Kcng2, Neurl2, Pltp, Tex14,* and *Vsig4* were normalized to *Rpl4* and expressed relative to non-stimulated cells **(Panels A-H respectively)**. * indicates significant differences P < 0.05. Values represent mean ± sem.

We next sought to use NEO to identify which of the module genes are directly downstream of *Cd44.* Thus*,* we repeated our NEO analysis with hepatic *Cd44* expression as the trait of interest. We used a LEO score of 0.3 as a threshold and predicted that 233 in the brown module genes are downstream of *Cd44*. Interestingly, of the 9 Hub genes, only *Fermt3, Trpv2* and *Plcg2* were predicted to be directly downstream of *Cd44*, indicating the effects of *Cd44* on the remaining 3 hub genes that are differentially expressed in *Cd44*^-/-^ macrophages are mediated by additional module members. In an attempt to further characterize the Brown module genes that may be responding to alterations in *Cd44*, we focused on validating the 9 additional genes with the highest LEO score (Table 3). To do so we first performed qPCR for the key module genes on thioglycolate-elicited peritoneal macrophages from *Cd44*^*-/-*^ and *Cd44*^*+/+*^ mice. We compared the expression of the brown module genes in unstimulated macrophages and observed small but statistically significant differences in *Kcng2, Dapp1, Neurl2, Fmnl2, Calhm2, Ehd4,* and *Pltp,* between *Cd44* and WT macrophages (Figure 6).

**Table 3:** **Genes predicted responsive to** ***Cd44***

**Gene symbol**	**Gene name**	**Chr**	**Mb**	**Leo score**	**% change Cd44**^**-/-**^**basal (P-value)**
**Kcng2**	Potassium voltage-gated channel, subfamily G, member 2	18	80	3.1	**NS**
**Dapp1**	Dual adaptor for phosphotyrosine and 3-phosphoinositides 1	3	137	3.08	**10% (0.05)**
**Neurl2**	Neuralized-like 2 (Drosophila)	2	164	2.99	**30% (0.005)**
**Fmnl2**	Formin-like 2	2	52	2.96	**17% (0.002)**
**Calhm2**	Calcium homeostasis modulator 2	19	47	2.7	**12% (0.03)**
**Ehd4**	EH-domain containing 4	2	120	2.67	**10% (0.008)**
**Pltp**	Phospholipid transfer protein	2	164	2.61	**18% (0.003)**
**Tex14**	Testis expressed gene 14	11	87	2.52	**-30% (0.02)**
**Vsig4**	V-set and immunoglobulin domain containing 4	X	96	2.5	**20% (0.03)**

### Network genes are differentially expressed in human atherosclerotic lesions

**Figure 7:**
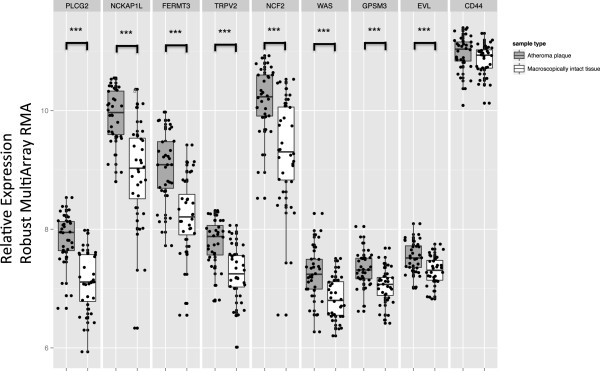
**Brown Module Hub Genes are differentially expressed in human atherosclerosis.** Publically available microarray data**,** GSE43292, was analyzed for differential expression of brown module hub genes, *Apbb1ip, Cd44, Evl, Fermt3, Gpsm3, Ncf2, Nckap1l, Plcg2, Tnf,* and *Trpv2*. Expression levels determined by robust multi-array normalization (RMA) for atherosclerotic sample (grey) and matched intact samples from the patients (white). * indicates significant differences P < 0.05. Values represent mean ± sem.

To better understand the overall translation of module to human atherosclerosis, we next sought to determine the expression of the module hub genes in human atherosclerotic tissue. We queried the Gene Expression Omnibus and identified GSE43292, a dataset where 32 hypertensive patients were treated for carotid atherosclerosis by endartectomy. The tissue from the patients was excised and advanced atherosclerotic tissue was compared to adjacent tissue without macroscopic evidence of atherosclerosis and both biopsies were subjected to expression array analysis. In this case all of our identified hub genes were differentially expressed between lesion and adjacent, normal tissue (Figure 7). There was no significant difference in *Cd44* expression between the macroscopically intact tissue and atherosclerotic tissue.

## Discussion

In the present study we use a network-based approach to interrogate the atherosclerosis susceptibility of the innominate artery. We identify 1 module that is highly related to IA atherosclerosis using liver tissue from a BxH *Apoe*^-/-^ genetic intercross in which we identified a major locus for IA atherosclerosis. This module is highly enriched for immune-related genes, in particular those of the monocyte/macrophage lineage. Overall, these studies have resulted in four main findings. First, we confirm that genes in this module are overexpressed in atherosclerotic tissue using publically available microarray data. Second, we demonstrate that the most connected genes in the module, “hub genes”, are differentially expressed in macrophages stimulated with *LPS* and between macrophages derived from C57BL/6J and C3H/HeJ mouse strains. Third, using structural equation modeling we predict that *Cd44* as the most likely candidate gene for our previously reported QTL and identify several genes predicted to be downstream of *Cd44.* Lastly, we validate novel interactions among the module genes using macrophages isolated from *Cd44*^*-/-*^ mice. Each of these findings is discussed below.

Our network analysis identified a module that was highly related to atherosclerosis in the IA and this module was highly enriched for genes in activated macrophages and spleen. Using freely available data at NCBI’s Gene Omnibus, we were able to confirm that the genes located in the brown module are significantly upregulated in the aorta of *Apoe*^-/-^ mice compared to age matched C57BL/6J mice. These results demonstrate that genetic networks identified in peripheral tissues, such as the liver, are able to identify biologically meaningful signals with relevance to disease.

None of the hub genes identified in the brown module have been previously linked to atherosclerosis. However, several of them are known to be involved in inflammation. For example, *Nckap1l*, also known as Hem1, was recently found to be an actin regulatory protein that interacts with *Was*, another of the hub genes in the brown module. *Was* has previously been shown to underlie Wiskott-Aldrich syndrome which is characterized by defective clotting and immune function and although *Was* has not been previously identified as a candidate gene for atherosclerosis, altered clotting function and immune function are thought to contribute to atherosclerotic lesion development. *Ncf2* is a causal gene for chronic granulomatous disease, while mutations in *Fermt3* lead to leukocyte adhesion deficiency, type 3. These data, along with reports of altered immune function in *Cd44*^-/-^ mice [21], indicate that interactions among these genes may affect atherosclerosis susceptibility and that this genetic pathway involves alterations in immune function.

In order to better understand how these genes respond to atherosclerosis related inflammatory signals, we stimulated peritoneal macrophages with *LPS*. These results indicated that the expression of several of these genes is significantly altered upon *LPS* stimulation. Furthermore, the expression of these differed between the strains of the original cross (C3H/HeJ and C57BL/6J). Together these results support our predicted genetic network. One potential difference between the original QTL study and the current network analysis and transcriptional validation study is a difference in the hyperlipidemic background of the mice. The original QTL was identified in hyperlipidemic *Apoe*^-/-^ mice which were originally developed to elevate lipids and sensitize them to atherosclerosis development [55] and several groups have used mice carrying this sensitizing mutation to identify QTL in mice [16,18,56,57]. We decided to use C3H/HeJ and C57BL/6J mice without the *Apoe*^-/-^ mutation based on the fact that the Chr 2 locus was independent of genetic signals for plasma lipids.

Our co-expression network is undirected and thus we cannot easily differentiate between causal and reactive genes for atherosclerosis in the brown module. Considering the known QTL for IA atherosclerosis on Chr 2 [15], we focused on genes which were located within the QTL boundary, and also present in the brown module. Only 11 genes of the Brown module genes are located within the 95% CI of the IA lesion size QTL (Additional file 3: Table S3). Our initial candidate gene highlighted in our previous publication was *Cd44* but we were unable to rule out the other 11 genes which reside in the QTL boundary, nor could we use the differential expression analysis to identify a single gene as 5 of the 11 genes located at the Chr 2 QTL and in the Brown module are differentially expressed in atherosclerotic tissue (*Cd44, Pamr1, Nusap1, Mertk,* and *Ehd4*).

Thus, we sought to use a bioinformatics approach called network edge orienting, which incorporates genotype data and structural equation modeling to determine causal genes within a gene list. The underlying approach has been validated for lipids and bone mineral density [24,35,43]. For this analysis we used all 666 brown module genes and found that only 1 gene, *Cd44,* was predicted to drive susceptibility to atherosclerosis. Therefore, we focused several experiments on understanding how *Cd44* may regulate expression of genes within brown module. To identify potential novel targets of *Cd44* within the brown module we used two approaches. First we identified which of the hub genes had an eQTL mapping to the atherosclerosis locus on Chr 2. The second approach repeated our NEO analysis using *Cd44* transcript levels as our phenotype of interest and the peak SNP for the atherosclerosis QTL, rs3671635, to anchor the analysis. Using these approaches we identified genes that are likely to be downstream of *Cd44*. To confirm these relationships we used peritoneal macrophages from *Cd44*^-/-^ mice and determined that expression of our predicted genes is indeed, modulated in part by *Cd44*. Thus, all of our genes with eQTL mapping to the atherosclerosis QTL interval *in trans*, *Nckap1l*, *Fermt3, Trpv2, Gpsm3* and *Evl*, were differentially expressed in *Cd44*^-/-^ mice. In addition to these genetic candidates, our NEO analysis identified over 100 genes in the module that are predicted to respond to *Cd44*. We tested 9 of these and confirmed that 8 of these are in fact reactive to *Cd44* expression levels.

Finally, we demonstrated that the brown module hub genes highlighted in this report are differentially expressed in human atherosclerotic tissue suggesting that the module of genes originally identified in the livers of atherosclerotic mice through gene co-expression analysis may, in fact, have a direct role in the development and progression of atherosclerosis. Interestingly, *CD44* was highly expressed in both the atherosclerotic and macroscopically intact adjacent tissue from humans and could indicate an important difference in the regulation of atherosclerosis in humans and mice. Alternatively, the lack of differential expression in the human tissues may reflect a high level of local vascular inflammation in the macroscopically intact tissues. This interpretation is supported by studies that indicate that *Cd44* is an early mediator of atherosclerosis [58] and is upregulated by pro-atherosclerotic cytokines [59]. Additional, studies are needed to clarify the role of *CD44* in atherosclerosis and differences between humans and mice in CD44’s role in regulating in the inflammatory response.

## Conclusion

We were able to identify 1 module of co-expressed genes that are related to atherosclerosis. This module contains our putative candidate gene and through a series of bioinformatics approaches we identify a potential mechanism for this QTL, through altered macrophage gene expression. None of these candidates has been previously identified as involved in atherosclerosis and thus represent novel targets for further investigation. Together these data demonstrate the utility of a network approach to prioritize genetic candidates and to identify potential mechanisms for candidate genes identified in QTL studies.

## Competing interests

The authors declare that they have no competing interests.

## Authors’ contributions

JA carried out the molecular *in vitro* experiments and assisted drafting the manuscript. PQ assisted with the *in-vivo* studies and assisted drafting the manuscript. AJL participated in the design of the study and assisted drafting the manuscript. BJB conceived of the study, and participated in its design, performed the statistical analysis and drafted the manuscript. All authors read and approved the final manuscript.

## Pre-publication history

The pre-publication history for this paper can be accessed here:

http://www.biomedcentral.com/1755-8794/7/51/prepub

## Supplementary Material

Additional file 1: Table S1 Module assignments for all network probes. This file contains the module assignments and annotations for each of the microarray probes contained in the bone co-expression network. Columns A–D provide probe annotations. Column E contains the connectivity score for each probe and column F is the module assignments. Click here for file

Additional file 2: Figure S1 Network dendrogram (top) and colors of modules (bottom). **Figure S2.** Topological overlap. Clustering with the topological overlap dissimilarity measure was used to identify gene coexpression modules, each of which was assigned a unique color. **Figure S3.** Connectivity is correlated with Gene Significance in brown module. **Figure S4.** Inflammatory response of Key Network Genes. **Figure S5.** Timecourse response to inflammation in Key Network Genes. Click here for file

Additional file 3: Table S2 An excel document with 3 tabs for enrichment analysis. Significant (FDR, 0.05) gene ontology enrichments for all 10 modules. This file is the output from DAVID and contains (FDR 0.05). More information about the output can be found at (http://david.abcc.ncifcrf.gov/). Click here for file
