# Supplementary material for: Genetic network identifies novel pathways contributing to atherosclerosis susceptibility in the innominate artery
Source: BMC Med Genomics. 2014 Aug 12;7:51. doi: 10.1186/1755-8794-7-51 (PMC4142055; doi:10.1186/1755-8794-7-51)
Supplement: Additional file 2: Figure S1 — Network dendrogram (top) and colors of modules (bottom). Figure S2. Topological overlap. Clustering with the topological overlap dissimilarity measure was used to identify gene coexpression modules, each of which was assigned a unique color. Figure S3. Connectivity is correlated with Gene Significance in brown module. Figure S4. Inflammatory response of Key Network Genes. Figure S5. Timecourse response to inflammation in Key Network Genes. [file 1755-8794-7-51-S2.docx]

**Supplementary Figure 1**: Network dendrogram (top) and colors of modules (bottom). Each line is an individual gene. Genes were clustered based on a dissimilarity measure (1 – TOM). The tips of the branches represent genes that are the least dissimilar and thus share the most similar network connections. Below dendrogram are colors that denote the module assignments. Gray indicates genes not assigned to a module.

Supplementary Figure 2: Topological overlap. Clustering with the topological overlap dissimilarity measure was used to identify gene coexpression modules, each of which was assigned a unique color. Rows and columns are symmetric and represent genes. The intensity of red color represents the absolute value of Pearson correlation coefficients. The rows and columns have been sorted by the gene clustering tree.

**Supplemental Figure 3.** Connectivity is correlated with Gene Significance in brown module. Plot showing the correlation (r=0.2, P=1.4x10^-12^) between connectivity and GS among Brown module genes.

**Supplemental Figure 4: Inflammatory response of Key Network Genes.** Peritoneal macrophages were isolated from C57BL/6J mice and treated in triplicate for 4 hours with increasing concentrations of *LPS.* Doses of *LPS* are indicated on the x-axis of each panel. Expression of *Apbb1ip, Cd44, Evl, Fermt3, Gpsm3, Ncf2, Nckap1l, Plcg2, Tnf, Trpv2,* and *Was* were normalized to *Rpl4* and expressed relative to non-stimulated cells (Panels A-K respectively). * indicates significant differences P<0.05. Values represent mean ± sem.

**Supplemental Figure 5- Timecourse response to inflammation in Key Network Genes.** Peritoneal macrophages were isolated from C57BL/6J mice and treated in triplicate for 4 hours with increasing concentrations of *LPS.* Doses of *LPS* are indicated on the x-axis of each panel. Expression of *Apbb1ip, Cd44, Evl, Fermt3, Gpsm3, Ncf2, Nckap1l, Plcg2, Tnf, Trpv2,* and *Was* were normalized to *Rpl4* and expressed relative to non-stimulated cells (Panels A-K respectively). * indicates significant differences P<0.05. Values represent mean ± sem.
